# Predictive Value of Cardiopulmonary Exercise Testing Parameters in Patients under Percutaneous Coronary Intervention with High Pulse Pressure

**DOI:** 10.31083/RCM25847

**Published:** 2025-02-18

**Authors:** Qiang Ren, Xingbo Mu, Yushan Li, Jian Zhang, Yanchun Liang, Quanyu Zhang, Yaling Han

**Affiliations:** ^1^State Key Laboratory of Frigid Zone Cardiovascular Diseases, Department of Cardiology, General Hospital of Northern Theater Command, 110016 Shenyang, Liaoning, China; ^2^Department of Cardiology, Beifang Hospital of China Medical University, 110016 Shenyang, Liaoning, China

**Keywords:** coronary artery disease, cardiopulmonary exercise testing, high pulse pressure, major adverse cardiovascular events

## Abstract

**Background:**

The correlation between cardiopulmonary exercise testing (CPET) parameters and the prognosis of patients undergoing percutaneous coronary intervention (PCI) with high pulse pressure (PP) is unclear. The purpose of present study is to investigate the correlation of CPET parameters in patients under PCI with high PP and assess their reference value for prognosis.

**Methods:**

Individuals aged 18 years and older who were diagnosed with coronary artery disease (CAD) and underwent PCI along with CPET from November 1, 2015 to September 30, 2021 were enrolled. The patients were categorized into two groups based on PP: high PP group (PP of males ≥50 mmHg; PP of females ≥60 mmHg) and normal PP group (PP of males <50 mmHg; PP of females <60 mmHg). The primary endpoint was major adverse cardiovascular events (MACE). The optimal predictors of MACE were identified through Cox regression analysis. The time-dependent receiver operating characteristic (ROC) curves were generated and the area under the ROC curve (AUC) was measured to evaluate the discriminatory ability in patients with high PP.

**Results:**

A total of 2785 patients were included in present study, with a median follow-up period of 1215 (687–1586) days. Through multifactorial analysis, it was determined that peak oxygen uptake (peak VO_2_, hazard ratio (HR): 0.94, 95% confidence interval (95% CI): 0.88 to 1.00, *p* = 0.038) and ventilatory equivalent for carbon dioxide (VE/VCO_2_, HR: 1.08, 95% CI: 1.02 to 1.15, *p* = 0.007) are important predictive factors in the parameters of CPET. The ROC based on diabetes mellitus (DM), smoking, peak VO_2_, and VE/VCO_2_ could effectively evaluate the prognosis of patients [1-year AUC: 0.636 (0.515~0.758), 3-year AUC: 0.675 (0.599~0.752), 5-year AUC: 0.718 (0.607~0.830)].

**Conclusions:**

The prognosis of CAD patients with high PP was worse compared to the patients with normal PP. The peak VO_2_ and VE/VCO_2_ were predictors of MACE in CAD patients with high PP.

## 1. Introduction

Coronary artery disease (CAD) is one of the most common cardiovascular diseases worldwide and remains a leading cause of death in both developed and developing countries [1, 2]. Additionally, percutaneous coronary intervention (PCI) is also a revolutionary progress in the treatment of coronary artery disease [3]. Several commonly used techniques, such as electrocardiography (ECG), routine non-invasive imaging tests, and non-invasive stress imaging [4], are currently accepted as effective methods for evaluating patients who have undergone PCI. However, it remains unclear which method should be used in clinical practice to evaluate the prognosis of this disease in specific situations. Compared to other invasive or non-invasive methods, cardiopulmonary exercise testing (CPET) is efficient, cost effective, and more convenient, uses no radiation for patients and does not require operators to undergo extensive professional training aiming [2]. In patients referred for chest pain, CPET demonstrated greater accuracy than traditional ECG stress testing in either identifying or excluding obstructive CAD [5]. Furthermore, CPET parameters such as oxygen pulse, peak oxygen uptake (peak VO_2_), maximal heart rate and ventilatory equivalents for carbon dioxide production (VE/VCO_2_) are closely related to cardiac prognosis [6, 7, 8, 9, 10]. Therefore, CPET provides objective, quantifiable, dynamic monitoring and non-invasiveness measurements of cardiac performance [11].

Pulse pressure (PP) is an index of arterial stiffening, measured as the D-value between systolic blood pressure (SBP) and diastolic blood pressure (DBP). As age increases, the elasticity of the arterial wall gradually weakens, and the pulse pressure increases, which can lead to an increase in left ventricular afterload, thereby adversely affecting the myocardial oxygen demand. This state may exacerbate myocardial ischemia in patients with coronary heart disease, thereby affecting their clinical outcomes. There is a growing body of evidence indicating that PP serves as an independent predictor of CAD risk among older and middle-aged individuals who have a heightened risk of cardiovascular disease [12, 13]. High PP is a negative prognostic factor for acute CAD and heart failure (HF), as reported by Haider* et al*. [14]. Therefore, further research is needed to identify the optimal parameters of CPET as predictors of adverse outcomes in patients with high PP.

The present study aims to find the optimal parameters of CPET for predictors of adverse outcomes in patients under PCI with high PP.

## 2. Materials and Methods

### 2.1 Study Design and Population

This single-center, retrospective, population-based study included consecutive patients aged over 18 years with CAD from November 1, 2015 to September 30, 2021. All data were regularly collected by a dedicated clinical research team using the ANYTHINK CV-NET clinical data collection system (Beijing Crealife Technology Co., Ltd. Beijing, China). Inclusion criteria: (a) admission for confirmed CAD; (b) at least 18 years old; (c) undergone PCI and received CPET within a week after PCI during hospitalization. Exclusion criteria include: (a) incomplete patient case information or follow-up information; (b) patients with chronic diseases such as chronic respiratory diseases or chronic liver and kidney failure; (c) patients with malignant tumors; (d) patients with abnormal mental states. The Medical Ethics Committee of General Hospital of Northern Theater Command approved the present study and waived consent provided by the patient [Y (2024) 097].

Previous research was established that a PP ≥50 mmHg in males and ≥60 mmHg in females is considered high PP [15]. Therefore, patients in present study were split into two groups according to their PP on admission: high PP group (PP of males ≥50 mmHg; PP of females ≥60 mmHg) and normal PP group (PP of males <50 mmHg; PP of females <60 mmHg).

### 2.2 CPET

CPET was conducted following PCI, with patients receiving standard medications [13]. Dynamic pulmonary function indicators were assessed using bicycle ergometers (SCHILLER, Baar, Switzerland). Baseline metabolic data were collected. Following a 3-minute rest period, a 3-minute duration of exercise without added resistance started at a rate of 60 revolutions per minute, followed by a steady rise in the load, lasting 8 to 12 minutes (progressively increased in accordance with the patient’s age, height, and weight by 10% of the expected exercise power [expected exercise power for males: 6.7730 + (136.141 * BSA) – (0.064 * age) – (0.916 * BSA * age); expected exercise power for females: 3.9330 + (86.641 * BSA) – (0.015 * age) – (0.346 * BSA * age); body surface area (BSA) = 0.007184 * weight^0.4250^ * hight^0.7250^]) [16]. Until the patient reaches volitional exhaustionor the test is terminated by the medical monitor [17]. After the procedure, the patient would rest for 10 minutes in the recovery phase, including 3 minutes of unloaded cycling, while the rehabilitation technicians would record the CPET test result [17].

The anaerobic threshold was determined based on the ventilatory equivalent for VO_2_ nadir while maintaining a consistent ventilatory equivalent (VE) for carbon dioxide production (VCO_2_) [18]. The relationship between VE and VCO_2_ was assessed by plotting VE against VCO_2_ values recorded every 10 seconds throughout the exercise period. The VE/VCO_2_ slope was derived using linear regression analysis on the ventilation and carbon dioxide production data collected during the entire exercise period, excluding the nonlinear part of the relationship after the onset of the acidotic drive to ventilation. Heart rate reserve (HRR) was calculated as the difference between maximum hazard ratio (HR) achieved with exercise and resting HR. Breathing reserve (BR) was calculated as the ratio of maximal ventilation during exercise to the maximum voluntary ventilation (MVV) at rest, both variables in L/min.

### 2.3 Clinical Data Collection and Follow-Up

The medical records of each included patient were obtained by retrospective review of clinical records and hospital computerized data. Baseline data included demographic information, medical history, clinical diagnosis, medications at discharge, imaging examination results, procedural information, laboratory indexes and CPET results.

In order to reduce measurement error, blood pressure (BP) in the right brachial artery was repeated twice, after a 5-minute break in the clinic, and then averaged. In addition, strenuous exercise was prohibited 1 hour before the BP measurement. Smoking, drinking strong tea and coffee were prohibited for 2 hours before the measurement, and there was a 2-minute interval between each measurement [19]. PP was measured and recorded after admission and before PCI. The calculation formula for PP is SBP on admission – DBP on admission. BP was measured and PP was calculated in accordance with the Japanese Society of Hypertension’s recommendations [20].

In the present study, clinical outcome data were obtained via telephone interviews. Follow-up was conducted at 1, 6, 12, 24, 36, 48, and 60 months, or until the study endpoint was achieved, or the trial was terminated.

### 2.4 Outcomes

The endpoint of the study was defined as the incidence of a major adverse cardiovascular event (MACE), including all-cause death, myocardial infarction (MI), and stroke. All-cause death occurred as a result of a cardiac event, unexplained sudden death, or a noncardiac cause. MI included acute myocardial infarction (AMI), coronary procedure-related MI and prior or silent/unrecognized MI. Stroke was sudden onset and rapid development of clinical signs including dizziness, weakness, speech difficulties, visual field defects, dysarthria, or other localized neurological impairments caused by vascular issues.

### 2.5 Statistical Analysis

Continuous variables were presented as mean ± standard deviation (SD) for normally distributed measurement data, while non-normally distributed data were shown as median (P_25_–P_75_). Categorical variables were represented by frequencies accompanied by percentages. Baseline data for the high PP group versus those in the normal PP group was compared by means of independent group Student’s *t*-tests, applying corrections for unequal variances as needed. When appropriate, categorical variables was compared by means of Chi-square tests or Fisher’s exact tests.

In the present study, univariate and multivariate Cox proportional hazards regressions were used to estimate HR and their 95% confidence intervals (95% CI). To evaluate the model’s discriminatory ability, the area under the time-dependent receiver operating characteristic (ROC) curve was used, with the corresponding area under the ROC curve (AUC) reported (0.5 = no information; 1.0 = perfect discrimination). A two-sided *p*-value < 0.05 was considered statistically significant. All statistical analyses were performed by using R version 4.1.2 (R Foundation for Statistical Computing, Vienna, Austria) and SPSS 27.0 (IBM Corp., Chicago, IL, USA).

## 3. Results

### 3.1 Study Population and Clinical Characteristics

**Fig. 1.**
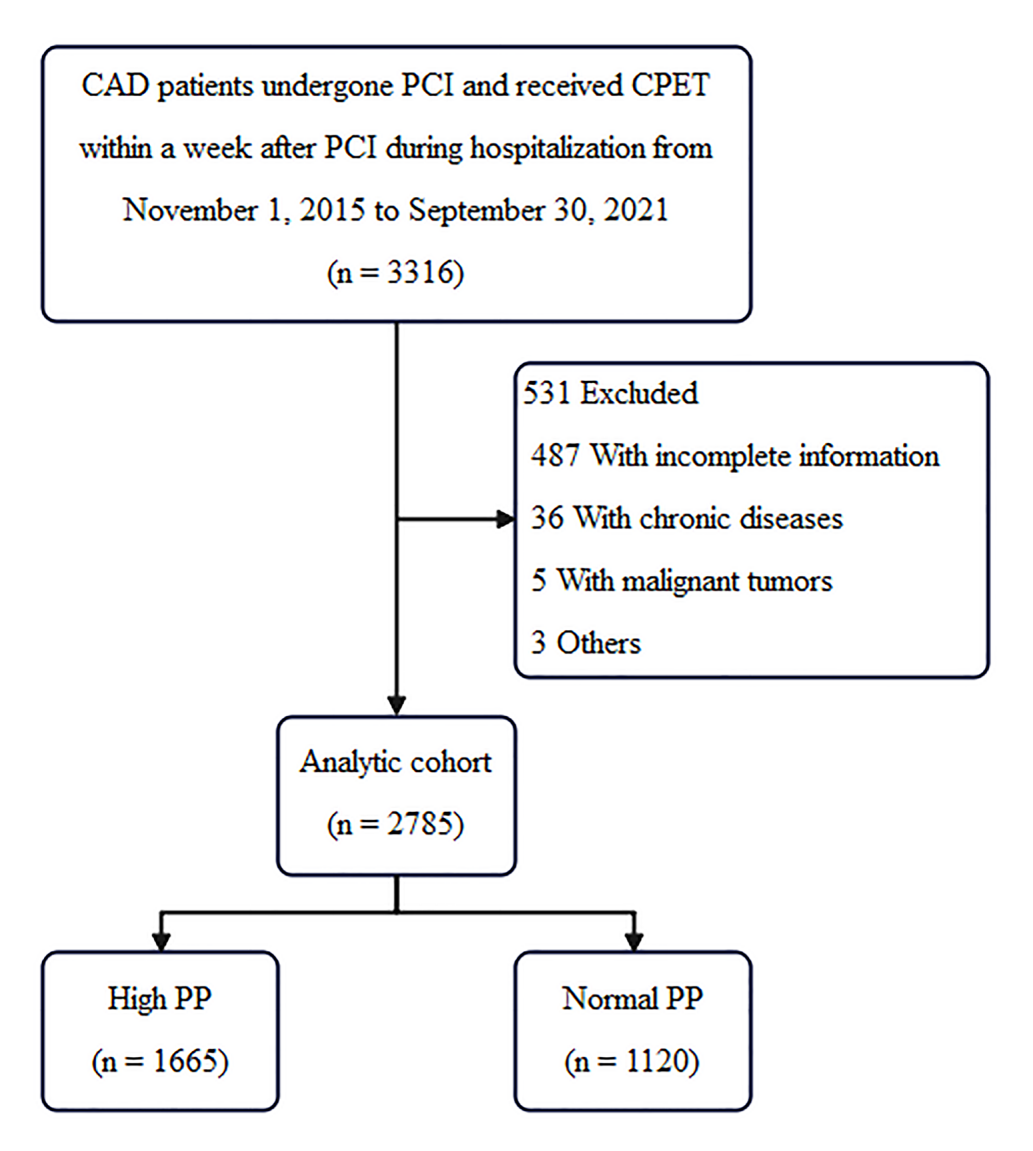
**Flow chart**. CAD, coronary artery disease; PCI, percutaneous coronary intervention; CPET, cardiopulmonary exercise testing; PP, pulse pressure.

Of the 3316 patients originally enrolled in the study, 487 patients were excluded for incomplete information. 36 patients were excluded for chronic diseases, 5 patients were excluded for malignant tumors, 3 patients were excluded for others. The results from the remaining 2785 patients were evaluated. A total of 2785 patients meeting the inclusion criteria were ultimately registered in the study (Flow chart in Fig. 1). Among them, 1665 (59.78%) patients met the high PP grouping criteria, PP in males ≥50 mmHg and PP in females ≥60 mmHg, were enrolled in the high PP group. 1120 (40.22%) patients were enrolled in the normal PP group.

Demographic information, medical history, indications for PCI, medication at discharge in both groups are shown in Table 1. The average age of all patients was 57.33 ± 8.35 years, and 79.89% were males. Proportion of males (82.94% *vs.* 75.36%, *p* < 0.001), proportion of hypertension (67.81% *vs.* 48.66%, *p* < 0.001), proportion of diabetes mellitus (DM, 31.35% *vs.* 23.13%, *p* < 0.001), previous stroke (11.53% *vs.* 9.11%, *p* = 0.048), SBP on admission (146.08 ± 15.61 mmHg* vs.* 123.48 ± 13.53 mmHg, *p* < 0.001) and left ventricular ejection fraction (LVEF, 61.43% ± 5.76% *vs.* 60.88% ± 6.18%, *p* = 0.018) were significantly higher in the high PP group compared with the normal PP group. The synergy between percutaneous coronary intervention with taxus and cardiac surgery (SYNTAX) score [11.00 (7.00, 16.13) *vs.* 12.00 (8.00, 17.00), *p* = 0.004], heart rate on admission (75.29 ± 11.73 beats/min *vs.* 77.73 ± 11.90 beats/min, *p* < 0.001) was lower in the high PP group. Patients in the high PP group had a higher use of ticagrelor (18.80% *vs.* 26.34%, *p* < 0.001), clopidogrel (81.14% *vs.* 73.57%, *p* < 0.001), calcium channel blockers (CCB, 31.53% *vs.* 22.86%, *p* < 0.001), angiotensin-converting enzyme inhibitors (ACEI, 27.87% *vs.* 21.01%, *p* < 0.001) and angiotonin receptor blocker (ARB, 34.95% *vs.* 29.11%, *p* = 0.001).

**Table 1.** **Baseline characteristics of patients**.

Clinical characteristics			Overall (n = 2785)	High PP (n = 1665)	Normal PP (n = 1120)	*p*-value
Age (years)			57.33 ± 8.35	57.54 ± 8.44	57.02 ± 8.22	0.106
Male, n (%)			2225 (79.89)	1381 (82.94)	844 (75.36)	<0.001
BMI (kg/m^2^)			25.51 ± 2.84	25.45 ± 2.78	25.59 ± 2.94	0.201
Smoking, n (%)			1139 (40.90)	702 (42.16)	437 (39.02)	0.098
Drinking, n (%)			671 (24.09)	405 (24.32)	266 (23.75)	0.728
Medical history, n (%)						
	Hypertension		1674 (60.11)	1129 (67.81)	545 (48.66)	<0.001
	Hypertension classification					0.817
		I	158 (5.67)	107 (6.43)	51 (4.55)	
		II	451 (16.19)	298 (17.90)	153 (13.66)	
		III	1081 (38.82)	732 (43.96)	349 (31.16)	
	DM		781 (28.04)	522 (31.35)	259 (23.13)	<0.001
	Previous PCI		793 (28.47)	469 (28.17)	324 (28.93)	0.663
	Previous stroke		294 (10.56)	192 (11.53)	102 (9.11)	0.041
	Previous MI		521 (18.71)	304 (18.26)	217 (19.38)	0.459
Heart rate on admission (beats/min)			76.27 ± 11.86	75.29 ± 11.73	77.73 ± 11.90	<0.001
SBP on admission (mmHg)			136.99 ± 18.50	146.08 ± 15.61	123.48 ± 13.53	<0.001
DBP on admission (mmHg)			81.06 ± 11.43	80.97 ± 11.26	81.18 ± 11.70	0.643
LVEF (%)			61.21 ± 5.94	61.43 ± 5.76	60.88 ± 6.19	0.018
Indications for coronary angiography, no. (%)						0.064
	Unstable angina		2199 (78.96)	1326 (79.64)	873 (77.95)	
	NSTEMI		324 (11.63)	202 (12.13)	122 (10.89)	
	Stable angina		2 (0.07)	1 (0.06)	1 (0.09)	
	STEMI		260 (9.34)	136 (8.17)	124 (11.07)	
SYNTAX score			12.00 (7.00, 17.00)	11.00 (7.00, 16.13)	12.00 (8.00, 17.00)	0.004
Medication at discharge, n (%)						
	Aspirin		2770 (99.46)	1657 (99.52)	1113 (99.38)	0.609
	Clopidogrel		2175 (78.10)	1351 (81.14)	824 (73.57)	<0.001
	Ticagrelor		608 (21.83)	313 (18.80)	295 (26.34)	<0.001
	Statin		2747 (98.64)	1647 (98.92)	1100 (98.21)	0.116
	β-Blocker		1739 (62.44)	1049 (63.00)	690 (61.61)	0.456
	CCB		781 (28.04)	525 (31.53)	256 (22.86)	<0.001
	Nitrates		1830 (65.71)	1103 (66.25)	727 (64.91)	0.467
	PPI		1292 (46.39)	771 (46.31)	521 (46.52)	0.913
	ACEI		700 (25.13)	464 (27.87)	236 (21.07)	<0.001
	ARB		908 (32.60)	582 (34.95)	326 (29.11)	0.001
	Diuretic		132 (4.74)	71 (4.26)	61 (5.45)	0.150
Laboratory indicators						
	Triglycerides (mmol/L)		1.42 (1.01, 1.98)	1.39 (1.00, 1.98)	1.45 (1.03, 1.98)	0.126
	LDL (mmol/L)		2.16 ± 0.77	2.16 ± 0.75	2.17 ± 0.80	0.728
	HDL (mmol/L)		1.08 ± 0.23	1.08 ± 0.23	1.08 ± 0.24	0.309
	Total cholesterol (mmol/L)		3.89 ± 1.06	3.88 ± 1.03	3.92 ± 1.10	0.392
	Troponin (ng/mL)		0.01 (0.01, 0.03)	0.01 (0.01, 0.03)	0.01 (0.01, 0.02)	0.085
	CKMB (U/L)		12.20 (10.00, 16.00)	12.00 (10.00, 16.00)	12.20 (10.00, 16.00)	0.163

Note: PP, pulse pressure; BMI, body mass index; DM, diabetes mellitus; PCI, percutaneous coronary intervention; MI, myocardial infarction; SBP, systolic blood pressure; DBP, diastolic blood pressure; LVEF, left ventricular ejection fraction; NSTEMI, non-ST elevation myocardial infarction; STEMI, ST elevation myocardial infarction; SYNTAX, the synergy between percutaneous coronary intervention with taxus and cardiac surgery; CCB, calcium channel blockers; PPI, proton pump inhibitor; ACEI, angiotensin converting enzyme inhibitor; ARB, angiotensin receptor blocker; LDL, low density lipoprotein; HDL, high density lipoprotein; CKMB, creatine kinase isoenzymes.

MACE occurred in 123 (4.41%) patients, including 44 (1.58%) patients with death, 39 (1.40%) patients with MI and 40 (1.44%) patients with stroke, over a median follow-up of 1215 (687–1586) days.

### 3.2 Effects of High PP on CPET Parameters

Parameters of CPET are shown in Table 2. Compared with the normal PP group, peak heart rate (116.91 ± 18.70 time/min *vs.* 119.98 ± 19.01 time/min, *p* < 0.001), forced expiratory volume in the first second (FEV_1_, 2.48 ± 0.63 L *vs.* 2.61 ± 0.68 L, *p* <0.001), MVV (102.97 ± 28.71 L/min *vs.* 107.36 ± 29.53 L/min, *p* < 0.001), peak oxygen pulse (9.55 ± 2.46 mL/beat *vs.* 9.82 ± 2.44 mL/beat, *p* = 0.004), breathing reserve (BR, 60.38% ± 12.16 % *vs.* 62.12% ± 11.66%, *p* < 0.001) were lower, while VO_2_ percent (56.04% ± 13.50% *vs.* 57.21% ± 13.24%, *p* = 0.024),VE/VCO_2_ (31.29 ± 3.72 *vs.* 30.97 ± 3.70, *p* = 0.025), SBPcpet (171.80 ± 28.39 mmHg *vs.* 160.10 ± 27.78 mmHg, *p* < 0.001), DBPcpet [84.00 (73.00, 92.00) mmHg *vs.* 82.00 (72.00, 91.00) mmHg, *p* = 0.017] was higher. There was no statistically significant difference among the other indicators (*p* > 0.05).

**Table 2.** **Comparison CPET in groups with normal and high pulse pressure**.

CPET parameters	Overall (n = 2785)	High PP (n = 1665)	Normal PP (n = 1120)	*p*-value
Peak heart rate (time/min)	118.15 ± 18.88	116.91 ± 18.70	119.98 ± 19.01	<0.001
FEV_1_ (L)	2.53 ± 0.66	2.48 ± 0.63	2.61 ± 0.68	<0.001
MVV (L/min)	104.74 ± 29.12	102.97 ± 28.71	107.36 ± 29.53	<0.001
VE (L/min)	39.13 ± 11.88	39.15 ± 11.68	39.09 ± 12.16	0.889
Peak power (W)	93.66 ± 28.18	93.64 ± 27.47	93.69 ± 29.22	0.965
Peak MET	4.42 ± 1.16	4.42 ± 1.11	4.42 ± 1.09	0.908
METAT	3.10 ± 0.59	3.11 ± 0.60	3.09 ± 0.59	0.332
Peak VO_2_ (mL/kg/min)	15.49 ± 3.87	15.50 ± 3.84	15.47 ± 3.92	0.867
VO_2_AT (mL/kg/min)	10.85 ± 2.08	10.88 ± 2.10	10.81 ± 2.06	0.333
VO_2_percent (%)	56.74 ± 13.36	56.04 ± 13.50	57.21 ± 13.24	0.024
Peak oxygen pulse (mL/beat)	9.71 ± 2.45	9.55 ± 2.46	9.82 ± 2.44	0.004
VE/VO_2_	29.28 ± 3.79	29.36 ± 3.75	29.17 ± 3.85	0.191
VE/VCO_2_	31.16 ± 3.71	31.29 ± 3.72	30.97 ± 3.70	0.025
SBPcpet (mmHg)	167.10 ± 28.72	171.80 ± 28.39	160.10 ± 27.78	<0.001
DBPcpet (mmHg)	82.00 (73.00, 91.00)	84.00 (73.00, 92.00)	82.00 (72.00, 91.00)	0.017
VE/VCO_2_ slope	27.62 ± 4.22	27.69 ± 4.07	27.51 ± 4.43	0.259
HRR (bpm)	44.34 ± 18.97	43.92 ± 18.76	44.97 ± 19.28	0.157
BR (%)	61.08 ± 11.99	60.38 ± 12.16	62.12 ± 11.66	<0.001

Note: PP, pulse pressure; CPET, cardiopulmonary exercise testing; FEV_1_, forced expiratory volume in the first second; MVV, maximal voluntary ventilation; VE, minute ventilation; MET, metabolic equivalent; METAT, metabolic equivalent anaerobic threshold; VO_2_, oxygen consumption; VO_2_AT, oxygen consumption anaerobic threshold; VO_2_percent, percentage of predicted peak VO_2_; VE/VO_2_, ventilatory equivalents for oxygen; VE/VCO_2_, ventilatory equivalents for carbon dioxide production; VE/VCO_2_ slope, slope of minute ventilation to carbon dioxide production; SBPcept, systolic blood pressure during CPET; DBPcpet, diastolic blood pressure during CPET; HRR, heart rate reserve; BR, breathing reserve.

### 3.3 Cox Regression Analysis of MACE in Patients with High PP

Univariate Cox analysis was used to analyze the relationship between clinical and CPET parameters and prognosis in patients with high PP. The univariate Cox analysis showed that DM, smoking, peak heart rate, peak power, peak metabolic equivalent (MET), metabolic equivalent anaerobic threshold (METAT), peak VO_2_, oxygen consumption anaerobic threshold (VO_2_AT), VE/VO_2_, and VE/VCO_2_ were significantly associated with patient prognosis (Table 3). Peak heart rate, peak power, peak MET, METAT, peak VO_2_, and VO_2_AT, were protective factors. DM, smoking, VE/VO_2_, and VE/VCO_2_ were risk factors. Consequently, they were included in the multifactor analysis, and the results showed that DM (HR: 1.75, 95% CI: 1.12 to 2.72, *p* = 0.015), smoking (HR: 1.61, 95% CI: 1.04 to 2.49, *p* = 0.033), peak VO_2_ (HR: 0.94, 95% CI: 0.88 to 1.00, *p* = 0.038) and VE/VCO_2_ (HR: 1.08, 95% CI: 1.02 to 1.15, *p* = 0.007) could be used as independent predictors of MACE (Table 3).

**Table 3.** **Univariable and multivariate Cox regression analysis in high PP patients**.

Characteristic	Univariate Cox regression analysis		Multivariate Cox regression analysis	
	HR (95% CI)	*p*-value	HR (95% CI)	*p*-value
Male	1.16 (0.64, 2.11)	0.609	-	-
Age (years)	1.02 (0.99, 1.04)	0.237	-	-
BMI (kg/m^2^)	1.05 (0.97, 1.14)	0.256	-	-
Hypertension	1.26 (0.77, 2.06)	0.339	-	-
DM (%)	1.84 (1.18, 2.85)	0.008	1.75 (1.12, 2.72)	0.015
Smoking (%)	1.57 (1.01, 2.43)	0.043	1.61 (1.04, 2.49)	0.033
Drinking (%)	0.96 (0.74, 1.24)	0.759	-	-
Triglycerides (%)	1.04 (0.87, 1.24)	0.701	-	-
LDL (mmol/L)	1.05 (0.79, 1.39)	0.745	-	-
HDL (mmol/L)	1.46 (0.59, 3.64)	0.416	-	-
Total cholesterol (mmol/L)	1.01 (0.82, 1.24)	0.954	-	-
Troponin (ng/mL)	0.81 (0.38,1.74)	0.531	-	-
CKMB (U/L)	1.00 (0.97,1.02)	0.691	-	-
Peak heart rate (time/min)	0.99 (0.97, 1.00)	0.019	-	-
FEV_1_ (L)	0.75 (0.53, 1.06)	0.103	-	-
MVV (L/min)	0.99 (0.99, 1.00)	0.096	-	-
VE (L/min)	0.98 (0.96, 1.00)	0.050	-	-
Peak power (W)	0.99 (0.98, 1.00)	0.007	-	-
Peak MET	0.71 (0.57, 0.87)	<0.001	-	-
METAT	0.6 (0.42, 0.88)	0.007	-	-
VO_2_percent	0.97 (0.96, 0.99)	0.002	-	-
Peak VO_2_ (mL/kg/min)	0.9 (0.85, 0.96)	<0.001	0.94 (0.88, 1.00)	0.038
VO_2_AT (mL/kg/min)	0.87 (0.78, 0.96)	0.007	-	-
Peak oxygen pulse (mL/beat)	0.92 (0.84, 1.01)	0.084	-	-
VE/VO_2_	1.09 (1.03, 1.15)	0.003	-	-
VE/VCO_2_	1.11 (1.05, 1.16)	<0.001	1.08 (1.02, 1.15)	0.007
VE/VCO_2_ slope	1.03 (0.98, 1.09)	0.237	-	-
SBPcpet (mmHg)	1.00 (0.99, 1.00)	0.469	-	-
DBPcpet (mmHg)	0.99 (0.97, 1.00)	0.099	-	-
HRR (bpm)	1.01 (1.00, 1.02)	0.079	-	-
BR (%)	1.00 (0.98, 1.01)	0.758	-	-

Note: PP, pulse pressure; HR, hazard ratio; 95% CI, 95% confidence interval; BMI, body mass index; DM, diabetes mellitus; LDL, low density lipoprotein; HDL, high density lipoprotein; CKMB, creatine kinase isoenzymes; FEV_1_, forced expiratory volume in the first second; MVV, maximal voluntary ventilation; VE, minute ventilation; MET, metabolic equivalent; METAT, metabolic equivalent anaerobic threshold; VO_2_, oxygen consumption; VO_2_AT, oxygen consumption anaerobic threshold; VO_2_percent, percentage of predicted peak VO_2_; VE/VO_2_, ventilatory equivalents for oxygen; VE/VCO_2_, ventilatory equivalents for carbon dioxide production; VE/VCO_2_ slope, slope of minute ventilation to carbon dioxide production; SBPcept, systolic blood pressure during CPET; DBPcpet, diastolic blood pressure during CPET; HRR, heart rate reserve; BR, breathing reserve.

**Fig. 2.**
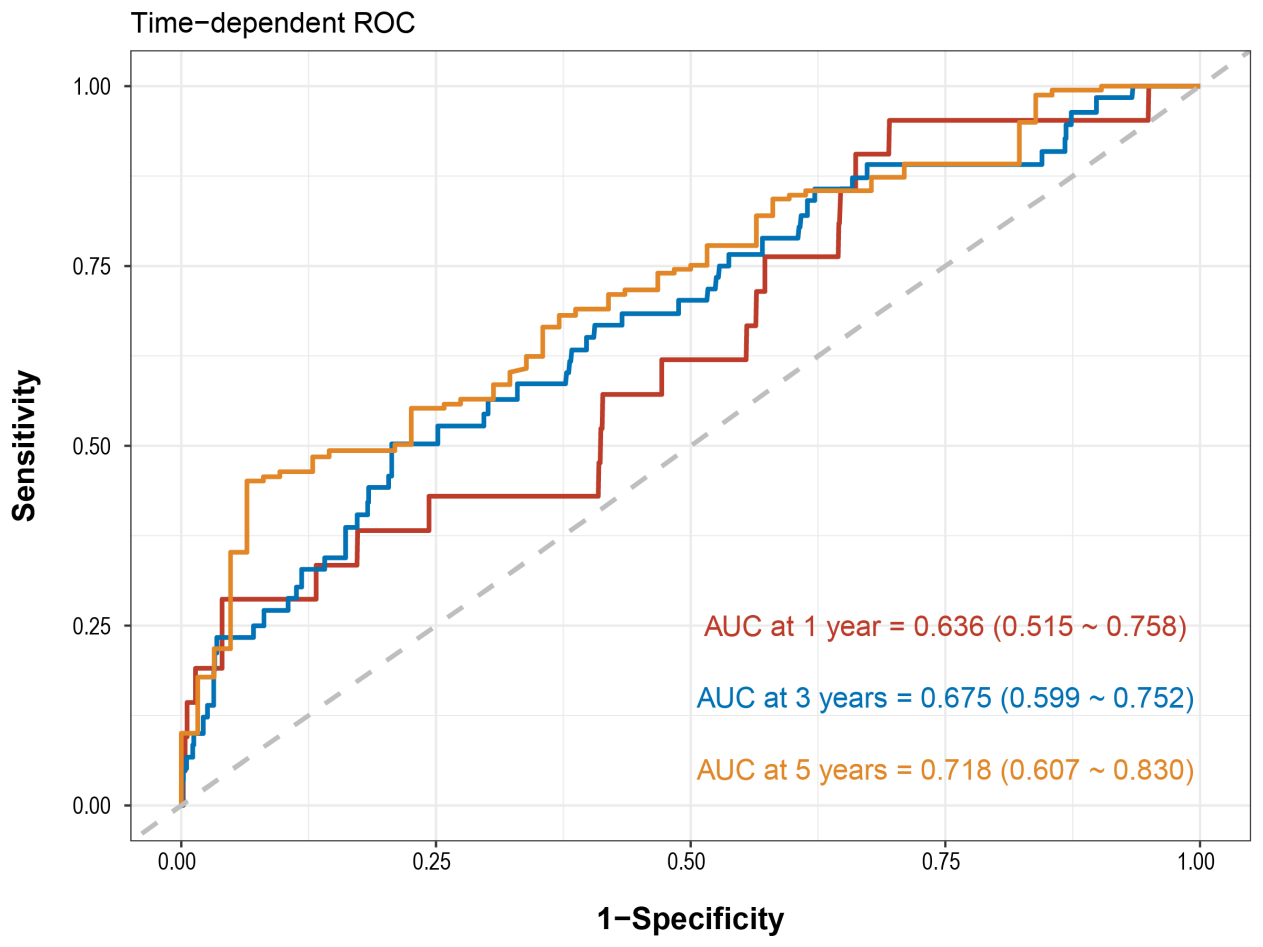
**Time-dependent ROC curve of predicting MACE among patients with high PP**. ROC, receiver operating characteristic; AUC, area under the ROC curve; MACE, major adverse cardiovascular events; PP, pulse pressure.

In the derivation cohort (DM, smoking, peak VO_2_, and VE/VCO_2_), the AUC for MACE over 1-year was 0.636 (0.515 to 0.758), while the 3-year AUC was 0.675 (0.599 to 0.752), and the 5-year AUC reached 0.718 (0.607 to 0.830), as illustrated in Fig. 2.

## 4. Discussion

In this retrospective analysis of high PP, we analyzed a total of 2785 patients with varying degrees of PP who underwent PCI for CAD. The median follow-up was 1215 (687–1586) days. The primary finding of present study was that patients with high PP had a worse prognosis compared to normal PP. In addition, we found that lower peak VO_2_ and higher VE/VCO_2_ were risk predictors of MACE in patients with high PP. These data suggests that in CAD patients with high PP, special attention should be paid to peak VO_2_ and VE/VCO_2_ during CPET.

CPET provides a non-invasive method to assess the cardiovascular, pulmonary, and skeletal muscle components of exercise performance. CPET is widely used to evaluate patients with systolic and diastolic heart failure, pulmonary hypertension, dilated cardiomyopathy, and congenital heart disease [21, 22, 23, 24]. The new findings of this study was the identification of the relationship between CPET results and the prognosis of patients with concomitant high blood pressure who have undergone PCI for CAD. These findings may be related to the increase in pulse pressure due to the decrease in aortic compliance. Previous study has shown that with aging, elastin degeneration and collagen deposition reduce the compliance and elasticity of conduit vessels [25]. This accelerates the transmission speed of the pulse wave within the arterial system, thereby shortening the time it takes for the forward pressure wave to travel from the aorta and peripheral arteries to various reflection sites and return to the heart. As a result, the reflected wave returns more quickly during systole rather than diastole, reaching the central arteries sooner, leading to an increase in central systolic pressure, a decrease in diastolic pressure, an enhancement of the central aortic pressure wave, and an increase in PP [26, 27, 28]. In the Strong Heart Study cohort, PP is independently associated with adverse cardiovascular outcomes [29]. Recent evidence indicates that elevated brachial PP is associated with adverse cardiovascular outcomes in high-risk atherosclerosis patients, independent of mean arterial pressure [30]. Furthermore, an elevated PP is also linked to chronic HF in older adults and can be considered a marker of high risk [14, 31]. Under normal arterial compliance, ventricular-vascular coupling during systole and diastole stores potential energy, converting the pulsatile cardiac ejection into continuous aortic blood flow. This maintains diastolic aortic pressure and improves coronary blood flow, thereby reducing left ventricular afterload. However, as arterial stiffening progresses, cardiac afterload increases because arterial wave reflections return more quickly during systole, raising systolic pressure, exerting additional stress on the left ventricle, and reducing coronary blood flow. This has been corroborated by the studies of Watanabe *et al*. [32] and Russo *et al*. [33]. In conclusion, the blood pressure response during exercise provides a window for assessing cardiovascular function in HF patients. In summary, based on the pathophysiological mechanisms of high PP and relevant findings of this study, the best CPET predictors of MACE in high PP patients are closely related to HF.

This study included patients with hypertension and CAD, which are major risk factors for the development and advancement of HF. These risk factors frequently coexist and have a synergistic effect [34, 35, 36, 37]. Identifying biomarkers to predict the prognosis of patients with cardiovascular disease remains a field that requires further exploration. Several studies focus on identifying additional parameters measured during CPET as potential prognostic indicators [38, 39]. VE/VCO_2_ and peak VO_2_ are the two predictive factors identified in this study, with VE/VCO_2_ reflecting pulmonary gas exchange and blood flow matching efficiency and exerting strong predictive abilities for both preserved and reduced ejection fraction HF [40]. Based on the alveolar gas equation, low arterial carbon dioxide partial pressure and abnormally high tidal volume dead space fraction, or the coexistence of both, can lead to an increase in the VE/VCO_2_ ratio [41]. In patients with HF, the range of arterial carbon dioxide pressure changes during exercise is smaller, which may be related to poor progression of arteriosclerosis or higher PP [42]. One of the characteristics of large-arterial stiffness is abnormal left ventricular systolic, which can lead to a reduction in cardiac output and, consequently, to an imbalance in ventilation and perfusion of the lungs. The current analysis indicates that VE/VCO_2_ has the highest ROC. Anuradha Lala *et al*. [40] corrected for circulatory power and peak Borg score, confirming that VE/VCO_2_ is the strongest predictor of advanced HF.

Peak VO_2_ reflects the maximal exercise capacity of patients. Although peak VO_2_ is not the optimal predictor of HF, it still has significant value as a reference parameter [43, 44], and in the diagnosis and prediction of myocardial ischemia. The diagnostic study by Belardinelli *et al*. [5] indicates that CPET has higher accuracy compared to ECG stress testing in evaluating myocardial ischemia in patients with chest pain. According to previous study, there is a significant negative correlation between peak VO_2_ and the prognosis of coronary heart disease [45]. Improving peak VO_2_ may have substantial benefits in reducing the burden of coronary heart disease [46]. Celutkiene *et al*. [47]. found that the peak VO_2_ of early heart failure with preserved ejection fraction (HFpEF) is associated with increased arterial stiffness. Due to arterial stiffness, arterial expansion is inhibited during left ventricular ejection, limiting blood storage in the arteries and the blood supply to peripheral tissues [48]. Additionally, it has been reported that the increased cardiac afterload caused by arteriosclerosis raises myocardial oxygen consumption, reduces cardiac output during peak exercise, and lowers cardiopulmonary function [49]. These factors collectively restrict VO_2_. Based on the analysis of this study, arterial stiffness is also a factor contributing to increased pulse pressure. Therefore, it can be inferred that high pulse pressure similarly affects peak oxygen uptake.

The present study found that age was not related to the incidence of MACE in the high PP group.Therefore, in CAD patients with high PP, any age has the same high risk of MACE. This observation had important implications for clinical practice. In the population with CAD, particular attention should be paid to the risk factors of CAD, including high PP. Regardless of age, CAD patients with high PP should receive increased attention and treatment when appropriate. The average age of population in the present study was 57.03 ± 8.90 years, which is lower than the population in previous studies [50]. Due to concerns about potential risks associated with CPET in elder patients, physicians in our center were conservative in recommending CPET. Previous studies have demonstrated that CPET is an efficient and safe method for CAD patients [51, 52], which benefits far outweigh their potential risks. In future clinical practice, physicians should be encouraged to adopt a proactive strategy towards conducting CPET on elderly patients.

In the present study, multivariate Cox regression analysis revealed that lower peak VO_2_ and higher VE/VCO_2_ were adverse factors for the long-term prognosis in the high PP group. Keteyian *et al*. [6] and Fujimoto *et al*. [53] reported that VE/VCO_2_ and peak VO_2_ were both significantly related to all-cause death and cardiovascular death in CAD patients. The difference from this present study may be attributed to the differences in patient enrollment. In previous study, there was a high proportion of patients with an AMI, while the present study enrolled patients undergoing elective PCI [6]. In summary, physicians should focus on peak VO_2_ and VE/VCO_2_ during CPET on patients undergoing elective PCI to accurately assess the prognosis of these patients.

The present study has several limitations: (a) This is a retrospective study, and there is a certain recall bias and selection bias. (b) The patients included in present study were younger compared to previous research. Therefore, our results need to be confirmed in CAD patients of all ages. (c) In present study, the proportion of male patients is relatively high, which may affect the generalizability of the results. Consequently, follow-up research should investigate other indicators derived from these parameters.

## 5. Conclusions

CAD patients with high PP generally have a worse prognosis. Additionally, lower peak VO_2_ and higher VE/VCO_2_ were risk predictors of MACE. Therefore, during cardiopulmonary CPET in these patients, special attention should be paid to peak VO_2_ and VE/VCO_2_ to assess cardiopulmonary function and guide clinical decisions, thereby optimizing treatment strategies and improving prognosis.

## Data Availability

The data sets analyzed during the current study are not publicly available due to restrictions apply to the availability of these data but are available from the corresponding author on reasonable request.

## Abbreviations

CAD, coronary artery disease; ECG, electrocardiograph; PCI, percutaneous coronary intervention; CPET, cardiopulmonary exercise testing; VO_2_, oxygen consumption; VE, minute ventilation volume; VE/VCO_2_, ventilatory equivalents for carbon dioxide production; PP, pulse pressure; SBP, systolic blood pressure; DBP, diastolic blood pressure; HF, heart failure; BSA, body surface area; MACE, major adverse cardiovascular events; MI, myocardial infarction; AMI, acute myocardial infarction; SD, standard deviation; HR, hazard ratios; BMI, body mass index; DM, diabetes mellitus; LVEF, left ventricular ejection fraction; NSTEMI, non-ST elevation myocardial infarction; STEMI, ST elevation myocardial infarction; SYNTAX, the synergy between percutaneous coronary intervention with taxus and cardiac surgery; CCB, calcium channel blockers; PPI, proton pump inhibitor; ACEI, angiotensin converting enzyme inhibitor; ARB, angiotensin receptor blocker; LDL, low density lipoprotein; HDL, high density lipoprotein; CKMB, creatine kinase isoenzymes; MET, metabolic equivalent; AT, anaerobic threshold; VE/VO_2_, ventilatory equivalents for oxygen; SBPcept, systolic blood pressure during CPET; DBPcpet, diastolic blood pressure during CPET; HRR, heart rate reserve; BR, breathing reserve.
